# Effect of Nucleic Acid Analog Administration on Fluctuations in the Albumin-to-Globulin Ratio in Cats with Feline Infectious Peritonitis

**DOI:** 10.3390/ani14091322

**Published:** 2024-04-28

**Authors:** Masato Katayama, Yukina Uemura, Daichi Katori

**Affiliations:** 1 Bloom Animal Hospital, Kajiyama 1-10-32, Tsurumi, Yokohama City 230-0072, Japan; marble1993.22@gmail.com; 2 Katori Animal Hospital, Migawa-cho 2563-16, Mito City 310-0913, Japan; daichi.katori@gmail.com

**Keywords:** cat, feline infectious peritonitis, non-effusive, nucleic acid analog, albumin-to-globulin ratio, feline coronavirus, routine treatment

## Abstract

**Simple Summary:**

A total of 122 and 56 cats with feline infectious peritonitis achieved remission after the administration of Mutian and molnupiravir (nucleic acid analogs with recently confirmed anti-viral effects) as routine treatments, respectively. Changes in various clinical indicators (body weight, hematocrit, and albumin-to-globulin ratio) before and after the administration of each drug and during follow-up observation were statistically compared for each of its three disease types (effusive, non-effusive, and a mixture of both). In all three disease types, the administration of either Mutian or molnupiravir resulted in statistically significant increases in the above three indicators. Furthermore, the effect of Mutian on improving the albumin-to-globulin ratio was not observed at all in the effusive forms, as compared with that of molnupiravir, but statistically significant in non-effusive and a mixture of both forms. The differences in the albumin-to-globulin ratio observed in the cats with non-effusive and mixed disease types were all due to differences in the fluctuations of circulating globulin levels, potentially indicating that slight inflammatory responses might be elicited continuously by residual feline coronavirus persisted through molnupiravir treatments.

**Abstract:**

Background: feline infectious peritonitis (FIP) is a fatal disease in cats classified as either effusive (‘wet’), non-effusive (‘dry’), or a mixture of both forms (‘mixed’). The anti-FIP therapeutic effects of Mutian and molnupiravir, two drugs with a nucleic acid analog as an active ingredient, have been confirmed recently. Methods: Of the cats with FIP, we observed a total of 122 and 56 cases that achieved remission after the administration of Mutian and molnupiravir as routine treatments, respectively. Changes in clinical indicators suggested to be correlated with FIP remission (weight, hematocrit, and albumin-to-globulin ratio) before and after the administration of each drug and during follow-up observation were statistically compared for each FIP type. Results: In all three FIP types, the administration of either Mutian or molnupiravir resulted in statistically significant increases in these indicators. Furthermore, the effect of Mutian on improving the albumin-to-globulin ratio was not observed at all in wet FIP, as compared with that of molnupiravir, but statistically significant in mixed and dry (*p* < 0.02 and *p* < 0.003, respectively). The differences in albumin-to-globulin ratio were all due to those of circulating globulin levels. Conclusions: These results indicate that slight inflammatory responses might be elicited continuously by a residual virus that persisted through molnupiravir treatments.

## 1. Introduction

Feline infectious peritonitis (FIP) is a fatal disease in cats caused by an exaggerated pro-inflammatory response to feline coronavirus (FCoV), and the cats infected with FCoV exhibit nonspecific clinical symptoms, such as recurrent fever, vomiting, and diarrhea, during the early stages of disease [1,2,3]. FIP is classified as effusive (wet), non-effusive (dry), or a mixture (mixed) of effusive and non-effusive forms [1,2]. The mixed type is a transitional state from one of the wet and dry types to the other, and shows symptoms of both types at the same time [1,3,4]. The wet type is characterized by fibrotic pleura–peritonitis with vasculitis and the exudation of body cavity fluid into adjacent areas, followed by the accumulation of exudate in the body cavities, including the abdomen, thorax, pericardium, and scrotum. The dry type is characterized by granulomatous lesions in several organs, including the central nervous system, and clinical signs in the eyes, whereas in the mixed type, features of both types are observed simultaneously [1,4]. Currently, the ante-mortem diagnosis of FIP is difficult, and there is no definitive noninvasive diagnostic test method for cats regardless of whether body cavity fluid can be collected, which is diagnosed comprehensively based on the veterinarian’s examination of physical signs, the results of various clinical tests, and viral RNA detection in routine veterinary care [3,5,6].

There are no treatments for FIP that have been legalized for clinical use outside countries such as the UK, and supportive care aimed solely at prolonging survival has been widely used for many years [7,8]. Additionally, several studies have suggested the possibility of treating FIP with immune-stimulants or other related drugs; however, no clinical benefit has been demonstrated [5,7,9,10]. In 2018, the nucleic acid analog GS-441524 was found to inhibit the replication of FCoV [11]. The prodrug GS-441524 (GS-5734, remdesivir) inhibits the replication of several taxonomically diverse RNA viruses [12,13]. Subsequently, the administration of GS-441524 to cats with spontaneous FIP and its therapeutic effects were confirmed; however, the initial method of administration was limited to subcutaneous injection [14,15]. An oral formulation of GS-441524 (designated as “Mutian X” in the later section) was initially developed by the Chinese company, making it available for both oral administration and subcutaneous injection routes [16,17,18]. Recently, with informed consent from the owners, we administered this drug to cats with FIP in our routine practice and investigated its effectiveness [19,20]. In these studies, remission rates of 82.3% (116 of 141 cases), 93.9% (153 of 163 cases), and 85.1% (137 of 161 cases) were observed in the wet type, dry type, and mixed-type FIP groups, respectively [19,20].

Additionally, molnupiravir (MPV), an orally administrable prodrug (EIDD2801) of N4-hydroxycytidine (EIDD1931) developed as an anti-COVID-19 drug, has been confirmed to exhibit antiviral activity against FCoV [21,22]. This drug has been approved in various countries as a treatment for human COVID-19 patients, and is now being used in veterinary care as a treatment for FIP in Japan. However, although many studies reported the use of Mutian as an FIP treatment, in contrast, very limited number of studies have described the use of MPV. Furthermore, there was no comparative analysis of its therapeutic effects on FIP.

In this study, we compared the changes in the clinical indicators via several laboratory tests for each case when each drug was used as a single agent for FIP treatment under routine veterinary care and analyzed the effects of both drugs on the remission of FIP and the quality of life of the cats according to the three disease types (wet, dry, and mixed). Furthermore, since both drugs routinely induce FIP remission, we investigated the differences in changes of clinical laboratory indicators, resultantly elucidating slight differences in their therapeutic efficacy, which may lead to contribute potential benefits to the cat’s owners. Therefore, to clarify statistical analysis, this study only included cases in which remission had been induced, and the cats for whom both drugs were used together were excluded even if administered temporarily.

## 2. Materials and Methods

### 2.1. Drugs and Administration

Orally administered Xraphconn (Mutian X) was purchased from Mutian Life Sciences (Nantong, China). The main active ingredient of Mutian X is demonstrated to be GS-441524 [18], and the manufacturer provides the information that the content of the main active ingredient in a 100 mg formulation is 5 mg [23]. Furthermore, the active ingredients in the subcutaneously administrative formulation (designated as “Mutian II”) are estimated to be the same as those in the orally administrative one, and may possibly be used interchangeably with Mutian X [20,24]; both were collectively designated as “Mutian” in the following section. These preparations were administered to cats with FIP, which was carried out according to our previous reports [19,20]. Briefly, Mutian was administered q24 h orally or subcutaneously for 12 weeks, and the dosages were increased or decreased in the range of 100–200 mg/kg, depending on each FIP type, and the disease stage. Mutian was administered orally or injected subcutaneously at a dosage of 100 mg/kg to the cats with wet-type FIP. Similar administration was performed for mixed-type FIP at the dosing of 150 mg/kg in the early-to-middle disease stages, or 200 mg/kg in the late stage. The dosing of 130 mg/kg in the early stage, 150 mg/kg in the middle stage, or 200 mg/kg in the late stage were performed for dry-type FIP [19,20]. Oral administration was performed by the cat owners. The temporary administration of the injectable form (Mutian II) required hospitalization at our facility; however, long-term administration was performed under the supervision of a veterinarian near the owner’s home.

Commercially available MPV powder capsules (200 mg/capsule) were purchased from Healing Pharma (Mumbai, India), Azista Industries (Hyderabad, India), or Mankind Pharma (New Delhi, India). The MPV capsule powder was completely dissolved in a simple syrup and orally administered to the cats. The veterinarian prescribed a dosage within the range of 10–20 mg/kg, considering the disease types or severity stages of FIP, based on twice-daily administration for 12 consecutive weeks, according to published reports using MPV as the first-line therapy for FIP [25].

### 2.2. Patients and Diagnostic Procedures

We enrolled 178 cats presented to our hospitals, which were diagnosed with FIP, received treatment with Mutian or MPV, were followed up, and ultimately achieved remission between December 2021 and December 2023. Notably, most of the fatal cases had already become seriously ill and died during the early stages of treatment, before the apparent therapeutic effects were observed. These cases were excluded since the study focused on the long-term effectiveness of the treatment drugs, and the essential requirement for all test values at four time points for approximately ≥100 days survival time and the onset of remission after treatments for statistical analysis.

Clinical specimens (whole blood with EDTA or plasma samples isolated by the centrifugation of heparinized whole blood) were collected at four time points: at the first dose, 1 month after starting the medication, after completion of each drug administration (84 days after the first dose), and 1–3 months after the completion of each medication. Body cavity effusions were collected from most cats with wet and mixed FIP. Total bilirubin (TB) and the albumin-to-globulin ratio (A/G) (DRI-CHEM4000V system, FUJIFILM Corporation, Tokyo, Japan), serum amyloid-A (SAA) (DRI-CHEM IMMUNO AU10V system, FUJIFILM Corporation), EDTA-treated whole-blood hematocrit (HCT) (MEK-6550 Celltac-α, NIHON KOHDEN Corporation, Tokyo, Japan), and α1-acid glycoprotein concentrations in plasma using the latex agglutination method (FUJIFILM VET Systems Co., Ltd., Tokyo, Japan) were also analyzed. Reverse transcriptase-PCR (RT-PCR) tests were performed by the Canine Lab Co., Ltd. (Tokyo, Japan) as previously described [19,20]. The circulating levels for total globulin were determined by subtracting those for the albumin from those for total protein according to the general biochemical calculation formula. The parameters of appetite (volume, frequency, and speed of feed intake) and activity (movement, walking speed, and agility) were assessed during the initial interviews with the owners according to the predetermined criteria and five interview items (classified into six stages 0–5), respectively. Each parameter was then converted into an arbitrary value as an appetite or activity score on a scale of 1–6; the scores were then analyzed statistically to assess the owner’s perception of the cat’s physical condition, along with the results of the routine physical examination at the clinic (body temperature, weight, ultrasonography, auscultation, and palpation). Ultrasonography was performed to determine the presence of ascites, pleural effusions, or swollen lymph nodes in the cats using the Aplio a CUS-AA000V ultrasound system (Canon Medtech Supply Co., Kanagawa, Japan).

The cats were diagnosed based on the same criteria as previously reported [19,20]. The disease characteristics were confirmed through a comprehensive examination of the apparent clinical signs (anorexia, underactivity, vomiting, diarrhea, seizures, tremors, ataxia, etc.), the qualitative PCR-based detection of FCoV in the blood, ascites, pleural effusions, and laboratory tests (HCT, whole cell count, total protein, TB, A/G, SAA, α1-acid glycoprotein, and activity of hepatic enzymes). The age, body temperature, and weight of all cats were recorded during the initial consultation with the veterinarian. The etiological ante-mortem diagnosis of FIP may be difficult, if not impossible, due to the invasiveness of biopsy collection from a sick cat, since there is currently no non-invasive confirmatory test available for cats without effusion [7]. Under such circumstances, FIP diagnosis was made by a comprehensive analysis of the above indicators. Diagnostic and treatment histories from other institutions, breeding status, birth, and adoption records, and the medical history of their previously cohabiting parents and siblings, were also recorded. In order to prevent subjective biases that might have favored the author’s theory construction, a nurse was required to help input information to the owner’s questionnaire and care was taken to exclude the veterinarian’s subjectivity. The cases used in our previous study were excluded from this study. Cases in which both Mutian and MPV were used in combination were excluded from this study.

Cats with FIP were classified as wet, mixed, or dry upon initial examination. The subsequent transition from wet to dry or from dry to wet conditions was not considered in this study. All cat owners were provided with detailed information regarding FIP treatment with Mutian and MPV, including the potential risks and benefits, estimated costs, and treatment duration. Written informed consent were obtained in advance from all owners regarding the selected treatment after mutual agreement between the veterinarians and owners. Additionally, both parties confirmed that Mutian or MPV were administered under optimal conditions to all cats as a standard of care and not as an experimental therapy. All data were obtained within the scope of normal veterinary practice and were appropriately anonymized. The studies were conducted in accordance with the local legislation and institutional requirements. The need for ethical review and approval was waived for these reasons.

### 2.3. Statistical Analysis

Age in months, appetite score, activity score, body temperature, and TB levels between the Mutian and MPV groups were statistically compared using the Mann–Whitney nonparametric U test. The Kolmogorov–Smirnov test was used to assess the normality of distribution for the measured variables. The statistical analysis of body temperature and circulating SAA levels between them were performed by unpaired *t*-test, as each of their data were judged to be normally distributed. Repeated-measure ANOVA was used to determine any significant change in the tested variables (body weight, HCT, and A/G) at four time points: at the time of the first dose, 1 month after starting the medication, after the completion of each drug administration (84 days after the first dose), and 1–3 months after the completion of each therapy. Repeated-measure ANOVA, accounting for the measurement of physical or clinical parameters (body weight, HCT, and A/G), treatment group (Mutian and MPV), and the interaction of the measurement and group, was used to determine and compare significant changes in the tested variables at the four time points in the groups. Differences were considered significant at *p* < 0.05. StatView 5.0 (SAS Institute, Cary, NC, USA) was used for statistical analysis.

## 3. Results

### 3.1. Baseline Characteristics of Observed Participants

The 178 cats with FIP in this study were classified into wet (57 cases), mixed (61 cases), and dry types (60 cases) according to the initial diagnosis by veterinarians (Table 1 and Table 2). All cats received the standard treatment with Mutian or MPV for 84 days. After the completion of the therapy, stable clinical signs with no obvious decline in the quality of life were continuously confirmed by the end of the 3-month observation period, and the cats were determined to be in remission.

Of the 57 cases with wet type FIP in this study, 43 were administered Mutian and 14 were administered MPV after receiving prior veterinarian consultation. Similarly, among the 61 cases of mixed-type FIP disease, 37 were treated with Mutian and 24 were treated with MPV. Furthermore, among all 60 cases of mixed-type FIP, 42 received Mutian treatment and 18 received MPV treatment (Table 1 and Table 2). For each type of FIP (wet, mixed, or dry), there was no statistically significant difference between the Mutian-administered and MPV-administered case groups in terms of age in months, appetite score, activity score, or TB value confirmed at the time of starting medication (Table 1). Based on the parametric statistical analysis (unpaired *t*-test), there were no significant differences in body temperature or SAA levels between the two groups, which were normally distributed parameters (Table 2).

### 3.2. Changes of Clinical Parameters over Times

Next, we examined the changes in the three parameters of body weight, HCT, and A/G values between four time points: the time of dosing each drug, 1 month later, the end of the standard dosing period, and their follow-up time point. Normal distributions of these three parameters in each group were statistically confirmed. In all disease type groups, statistical significance was confirmed in the variation patterns of each index between the four time points due to Mutian administration and MPV administration in body weight, HCT, and A/G values. The results of the repeated-measure ANOVA showed that treatment with both drugs effectively changed all three indicators for all wet, mixed, and dry types (*p* < 0.0001).

**Figure 1:**
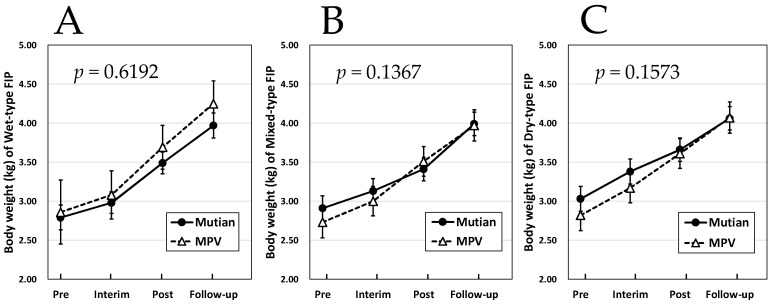
Changes in body weight in Mutian and MPV administration groups in the cat with wet-type (**A**); mixed-type (**B**); and dry-type FIP (**C**). Statistically significant differences between treatments (Mutian and MPV) for the effects on each parameter were determined using repeated-measure ANOVA. Of the 57 cases with wet-type FIP, 43 were administered Mutian and 14 were administered MPV. Among the 61 cases of mixed type, 37 were treated with Mutian and 24 were treated with MPV, and among the 60 cases of dry type, 42 received Mutian and 18 received MPV treatment. The cats were tested at four time points: at the first dose (Pre); 1 month after starting the medication (Interim); after the completion of each drug administration (Post); and 1–3 months after the completion of each medication (Follow-up). Symbols and vertical lines in the graph indicate mean values and standard errors, respectively. Abbreviations: MPV: molnupiravir; FIP: feline infectious peritonitis.

**Figure 2:**
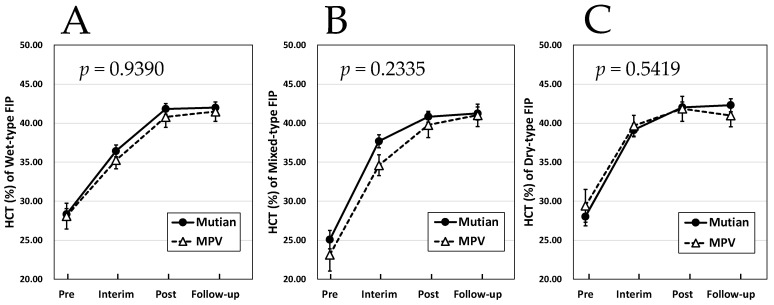
Changes in HCT in Mutian and MPV administration groups in the cat with wet-type (**A**), mixed-type (**B**), and dry-type FIP (**C**). Statistically significant differences between treatments (Mutian and MPV) for the effects on each parameter were determined using repeated-measure ANOVA. Of the 57 cases with wet-type FIP, 43 were administered Mutian and 14 were administered MPV. Among the 61 cases of mixed type, 37 were treated with Mutian and 24 were treated with MPV, and among the 60 cases of dry type, 42 received Mutian and 18 received MPV treatment. The cats were tested at four time points: at the first dose (Pre), 1 month after starting the medication (Interim), after completion of each drug administration (Post), and 1–3 months after completion of each medication (Follow-up). Symbols and vertical lines in the graph indicate mean values and standard errors, respectively. Abbreviations: HCT, hematocrit; MPV: molnupiravir; FIP: feline infectious peritonitis.

**Figure 3:**
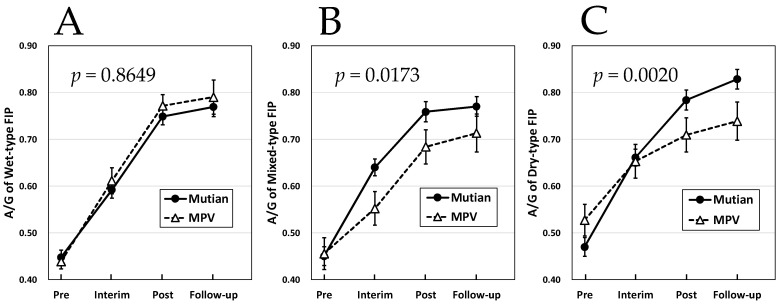
Changes of A/G in Mutian and MPV administration groups in the cat with wet-type (**A**), mixed-type (**B**), and dry-type FIP (**C**). Statistically significant differences between treatments (Mutian and MPV) for the effects on each parameter were determined using repeated-measures ANOVA. Of the 57 cases with wet-type FIP, 43 were administered Mutian and 14 were administered MPV. Among the 61 cases of mixed type, 37 were treated with Mutian and 24 were treated with MPV, and among the 60 cases of dry type, 42 received Mutian and 18 received MPV treatment. The cats were tested at four time points: at the first dose (Pre), 1 month after starting the medication (Interim), after the completion of each drug administration (Post), and 1–3 months after completion of each medication (Follow-up). Symbols and vertical lines in the graph indicate mean values and standard errors, respectively. Abbreviations: A/G: albumin-to-globulin ratio, MPV: molnupiravir; FIP: feline infectious peritonitis.

Figure 1 and Figure 2 showed that no interaction was observed between the case groups treated with these two drugs in terms of increase in body weight and HCT for any disease type, and there was no statistically significant difference in the effects of the two drugs. Furthermore, regarding the course of the increase in A/G, the effects of both Mutian and MPV were not significantly different for the wet FIP (Figure 3A). However, an interaction between the two drugs was observed in either of the mixed or the dry FIP, and a statistically significant difference was detected between the two drugs in terms of their effects on the increasing trend of A/G as shown in Figure 3B,C (*p* < 0.02 or *p* < 0.003, respectively). In addition, their increasing trends in the A/G appeared to be different throughout, from initiation to the end of the administration period (Figure 3B,C).

### 3.3. Changes of Albumin and Globulin Levels in Circulation over Times

In order to investigate a key factor which has induced the phenomenon that the improvement trend of A/G in dry- and mixed-type FIP cases was statistically different between two treatments, we tried to analyze the fluctuations of their albumin and globulin levels separately.

**Figure 4:**
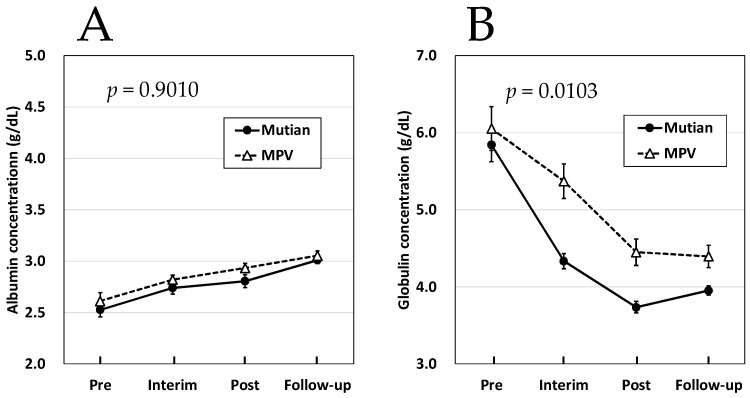
Changes of albumin (**A**) and globulin concentration (**B**) in Mutian and MPV administration groups in the cat with mixed-type FIP. Statistically significant differences between the treatments (Mutian and MPV) for the effects on each parameter were determined using repeated-measure ANOVA. Among the 61 cases of mixed type, 37 were treated with Mutian and 24 were treated with MPV. The cats were tested at four time points: at the first dose (Pre), 1 month after starting the medication (Interim), after the completion of each drug administration (Post), and 1–3 months after completion of each medication (Follow-up). Symbols and vertical lines in the graph indicate mean values and standard errors, respectively. Abbreviations: MPV: molnupiravir; FIP: feline infectious peritonitis.

**Figure 5:**
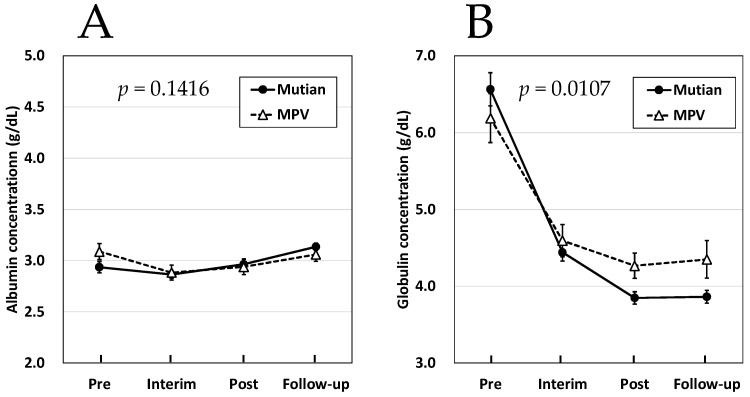
Changes of albumin (**A**) and globulin concentration (**B**) in Mutian and MPV administration groups in the cat with dry-type FIP. Statistically significant differences between the treatments (Mutian and MPV) for the effects on each parameter were determined using repeated-measures ANOVA. Among the 60 cases of dry type, 42 were treated with Mutian and 18 were treated with MPV. The cats were tested at four time points: at the first dose (Pre), 1 month after starting the medication (Interim), after the completion of each drug administration (Post), and 1–3 months after completion of each medication (Follow-up). Symbols and vertical lines in the graph indicate mean values and standard errors, respectively. Abbreviations: MPV: molnupiravir; FIP: feline infectious peritonitis.

Regarding the course of the increase in albumin concentrations, the effects of both Mutian and MPV were not significantly different for the mixed FIP (Figure 4A). However, an interaction between the two drugs was observed for globulin levels in the mixed FIP, and a statistically significant difference was detected between the two drugs in terms of their effects on the increasing trend of globulin concentrations, as shown in Figure 4B (*p* < 0.02). In dry-type FIP, almost no changes in albumin concentrations along with the course of both treatments has been detected (Figure 5A). As comparable with those in the mixed type, however, a statistically significant difference was detected between the two drugs in terms of their effects on the increasing trend of globulin concentrations (Figure 5B, *p* < 0.02).

## 4. Discussion

Regarding the changes in the three parameters of body weight, HCT, and A/G values between the two time points of dosing each drug and the end of the standard treatment process, at least the changes in each parameter due to the Mutian formulation, we observed a significant increase in body weight, HCT, and A/G levels (Figure 1, Figure 2 and Figure 3), similar to our previous reports [19,20]. Additionally, it was confirmed that both Mutian and MPV had statistically significant effects on the changes in each parameter between the four time points, including the follow-up time after the end of medication. Although our current study was conducted under the conditions of general practice and with an extremely limited number of cases, this is the first study in which a comparative analysis of the time series was conducted using two different types of anti-FCoV nucleic acid analogs to evaluate their pharmacological effects in parallel.

Notably, this study might have entailed some biases, particularly regarding case selection for comparing the clinical effects of two distinct therapeutic drugs. However, since the purpose of the study was to compare their efficacy within the scope of routine medical treatment, we comparatively analyzed physical characteristics including disease severity. It was also necessary to ensure that the treatment groups did not differ significantly regarding physical and clinical conditions, including disease severity, to minimize the influence of these biases.

FIP is recognized as a disease that primarily affects cats aged between 6 months and 2 years of age [26]. It is also recognized that the initial diagnosis of this disease is based first and foremost on signalments, including the cat’s age, place of birth, clinical signs, physical examination, and breeding status for cats between 4 and 36 months of age in group housing. In this study, we investigated the ages of all cats with FIP and analyzed whether there were any differences between the case groups. We referred to existing information obtained from the initial diagnosis of FIP and showed that the measured body temperature may be a useful numerical index for predicting the effect of Mutian treatment on subsequent FIP disease, in addition to the appetite and activity scores calculated based on the results of interviews with owners at the time of the first diagnosis in the previous studies [19,20].

Hyperbilirubinemia and bilirubinuria commonly occur in FIP, and both have no correlation with the blood concentrations of liver dysfunction indicators and are known to occur particularly at high frequencies in effusive FIP [7]. The previous studies demonstrated that the TB concentration at the time of initial diagnosis is a useful clinical index for predicting the subsequent therapeutic effect of Mutian and that this phenomenon is limited to effusive FIP [19,20]. Furthermore, as an acute phase protein, SAA is known to be normally almost undetectable and increase with viral infections that cause acute systemic inflammation. Elevated SAA levels may facilitate the early and effective detection of many viral infections such as FIP, since it strongly mirrors clinical conditions [27]. Although these six clinical or physical numerical indicators, including age, may be correlated with the prognosis of the therapeutic effect of the nucleic acid analog preparation, no statistically significant differences were detected between the parameters of the two treatment groups, and no significant differences were observed for each FIP type in the present study (Table 1 and Table 2). Considering the above results, we consequently judged that there was no noteworthy difference in FIP severity or physical condition upon their first visit to our hospital, which may affect the effectiveness of subsequent drug treatment, between cats whose owners selected the administration of Mutian and MPV.

In the present study, although not observed in wet-type FIP, a different effect of the two drugs on the A/G increase was observed in each of the mixed and dry type, and the difference was confirmed to be apparently significant (Figure 3B and Figure 3C, respectively). In the four-time series period of the Mutian and MPV treatment groups for dry-type disease, the tendency for an increase in A/G after the end of the treatment period was clearly different between the two drugs, and a significant difference was observed in the period from the end of treatment to follow-up observation (Figure 3C). One retrospective study in China previously indicated critical value of the A/G could be considered as 0.5, FIP was negligibly detected when the A/G > 0.8 and its possibility remained within those ranges [3,26,28,29]. In our present study, even if observed in the mixed and the dry FIP cases, their A/G reached around 0.8 only at Mutian administration, suggesting the disease remission might be more evident (Figure 3B,C). These data led to some possible assumptions as follows: either the therapeutic effect of Mutian on dry-type FIP is superior to that of MPV, MPV has any independent negative effects (including unknown adverse effects or irregular pharmacokinetics) of its own, or both occur simultaneously.

Because MPV has been confirmed to have medicinal efficacy in treating coronavirus infections other than FCoV, some owners have used commercially available MPV to treat cats with FIP at home. However, there have been extremely limited reports that scientifically verified the clinical effects of MPV on FIP [30]. One previous report suggested that it is desirable to use MPV as an emergency treatment when disease symptoms persist or recur after a cat with suspected FIP is treated as the first-line therapy using drugs with proven track records, such as GS-441524 [25]. In the other study, several cases were confirmed that exhibited nausea symptoms even with the standard dose of MPV (10 mg/kg, twice a day); when the dose was further increased (23 mg/kg, twice a day), various side effects (ear breakage, hair loss, and severe leukopenia) have been observed with multiple administrations of MPV [25,31]. Furthermore, unlike the wet type, the dry and mixed types of FIP are characterized by granulomatous lesions in various organs of the body, ocular symptoms, and neurological symptoms [1,5,6,7]. It has been confirmed that the anti-FCoV agent GS-441524, MPV, and its activated form N4-hydroxycytidine (EIDD1931) can cross the blood–brain barrier and migrate into the brain after administration into the blood. However, because the transfer efficiency from circulation to intraocular tissues and cranial nerves is not sufficient, it is necessary to increase the dosage to obtain a sufficient amount of the drug [11,32,33]. In our study, we included cases in which the dosage of MPV was increased to a maximum of 20 mg/kg twice a day, depending on the symptoms of each type of FIP; however, higher dosages were not always observed in non-effusive FIP The relationship between the phenomenon observed here and the abovementioned issues reported with MPV requires further research on the following hypothesis; individual differences in the pharmacokinetics of MPV administered to cats may potentially lead to low efficiency in the active ingredient leach to granulomatous tissues characteristic of mixed or dry FIP, possibly causing virus persistence in some cases as a result.

Three disease types of FIP (wet, mixed, and dry types) are presumed to occur because of the status of the host defense immunity against FCoV [1]. Macrophages infected with FCoV acquire the ability to destroy viruses; however, infected macrophages can be recognized as foreign substances by the host immune system and are further destroyed. If cell-mediated immunity is well developed early in the infection process, clinical signs of FIP will not occur and viral replication will be inhibited; however, if humoral immunity occurs but cell-mediated immunity does not develop; wet type FIP develops resultantly. However, an intermediate stage of immunity involving strong humoral and weak cellular immunity may also occur simultaneously, resulting in the development of a mixed type. If the equilibrium between humoral and cell-mediated immunity persists, a dry-type FIP develops [1]. In all disease types, an increase in the concentration of γ-globulin, which is mainly composed of antibodies in serum proteins, accompanying the enhancement of humoral immunity is a clinical chemical feature commonly observed in all of the FIP cats [26,34]. Most cats with FIP present with abnormalities in serum biochemistry, primarily hyperproteinemia, hyperglobulinemia (even in the absence of increased serum total protein), and hypoalbuminemia. Furthermore, among these biochemical abnormalities, hyperglobulinemia has the highest frequency at 89% [26,34]. Our present study revealed that the discrepant fluctuation of A/G levels between Mutian- and MPV-administered FIP cases is due to the differences in reducing the tendency of circulating globulin concentrations in them (Figure 4 and Figure 5). We can speculate, therefore, that insufficiently reduced levels of circulating globulin (mainly defined as immunoglobulin) possibly indicate any residual infective agents retained in the MPV-administered cats. Although MPV has been the first oral antiviral authorized for COVID-19, however, it has recently been reported to be associated with poor clinical efficacy, the risk of creating novel coronavirus variants of concern, and long-term risk for mutagenicity in humans [35]. The former two are severe concerns, especially in the FIP treatments considered in the current study.

The first limitation of our present study was that this study appears to be insufficient as a formal test for evaluating the clinical effectiveness of a new therapeutic agent. In the initial step of evaluating the clinical efficacy of a new drug, it is desirable to conduct a clinical trial based on case selection using a random sampling method, such as a randomized placebo-controlled clinical trial. First, there are no officially approved drugs or treatments for FIP, and it is difficult to establish a comparative therapy or positive control drug. A blinded non-inferiority study to compare the clinical effects of GS-441524 and remdesivir on FIP has recently been conducted, and the authors have also reported that the initiation of medication was delayed because of the allocation and long-distance transport of the cats, resulting in lower remission rates for both therapies compared to other previous reports [36]. Unfortunately, setting up such a controlled clinical trial seems difficult because our top priority in routine clinical practice is to save each cat’s life. The results of this non-inferiority trial indicate that it may be important to ensure the success rate of prospective treatment to gain owners’ attendance.

The second limitation of this study was that the population of the cat FIP group used for comparison was slightly small; in particular, the number of cases in which MPV was administered as a first-line drug was extremely small (14 to 24 cases). Furthermore, in this study, we compared the physical signs and disease severity before treatment between the Mutian and MPV administration groups to maximize our ability to confirm the equivalence between these two groups, but it is difficult to deny that there may be any bias in the selection of cases in either of these groups with respect to determining the effectiveness of the drugs. For example, because MPV is limited to oral administration, cases in which oral administration is difficult due to gastrointestinal disorders must be automatically excluded. In contrast, Mutian can be administered both subcutaneously and orally; therefore, cats with possible gastrointestinal disorders are not usually excluded, resulting in the fact that this type of bias does not occur because Mutian can be administered subcutaneously to cats that are unable to swallow. In the future, the manufacturers and distributors of each drug should take the lead in taking these points into account and set appropriate conditions, especially in case selection, the planning, including the evaluation of clinically parameters (such as time to remission, relapse rates, adverse effects), and the execution of clinical trials, leading to their formal approval from the authorities.

The third limitation involved potential ethical and legal issues. First, the uncontrolled expansion of the utilization of antiviral agents may accelerate the FCoV mutation itself, which in turn may lead to the generation of new infectious viruses that are potentially harmful to humans. We previously stated that the widespread use of antiviral drugs as preemptive therapy does not benefit the public as it increases the risk of multi-drug resistance and epidemic infections [19]. We cannot recommend the widespread use of such anti-FIP drugs without veterinary supervision, or that the cat’s owners without medicinal knowledge voluntarily obtain these drugs by means of online shopping and dose them to their cats at home. We also strongly discourage the owner-driven, autonomous practice of administering any medicines to pets, and instruct owners who visit our hospital to always administer medication under veterinarian’s consultation and supervision. Of course, we only administered anti-FIP drugs to the cases suspected as FIP through our diagnosis, and ensured that the benefits to the cases and their owners outweighed the risks. In this study, only the qualitative detection of a residual virus in bodily fluids and feces was performed using PCR. Unfortunately, PCR tests are currently outsourced to external organizations and are only qualitative tests unable to detect all virus mutations. We recognize that molecular biology-based verification may be very important in detecting FCoV mutations, and look forward to future scientific progress by specialists for detecting resistant viral mutations caused by antiviral drugs. Regarding legal issues, under the current legal framework in Japan, the use of any unauthorized veterinary drugs as therapeutics is recognized within the judgment and discretion of individual veterinarians with the prior consent of pet owners. Furthermore, we consulted a lawyer in Japan regarding this issue and confirmed that there were no legal issues.

Recently, a long-term prognostic follow-up study for 1 year after the confirmation of remission after the treatment of FIP with Mutian X (orally administered GS-441524) was conducted, demonstrating its effectiveness against FIP in both the short and long term, with no significant relapse [37]. Considering the results of our study and those of previous reports, oral GS-441524 may still be preferable as a first-line drug for FIP therapy. MPV should be considered for use in cats if Mutian’s efficacy is not expected, but veterinarians should be careful when increasing the MPV dosage.

## 5. Conclusions

In this study, we confirmed significant improvements in the physical and clinical laboratory indicators for each type of FIP by administering Mutian or MPV, which have already been reported as drugs for the treatment of FIP, and confirmed clear therapeutic effects. The statistical analysis of transitional changes in the clinical parameters (body weight, HCT, and A/G levels) suggested to be correlated with FIP remission before and after each drug administration, and at subsequent follow-up observations for each type of FIP group revealed a significant difference in the effects of both drugs on A/G transition fluctuations, especially in cats with mixed- or dry-type FIP. The clinical features observed in these cases were all due to differences in fluctuations of circulating globulin levels, indicating that host immuno-responses might be induced by residual FCoV after MPV treatments. This phenomenon confirmed this time may help us elucidate the relationship between the granulomatous inflammation in several organs of non-effusive FIP, the tissue distribution of antiviral therapeutics and A/G fluctuations, as well as the future directions of developing new nucleic acid analogs effective for FCoV.

## Figures and Tables

**Table 1:** Nonparametric statistical comparison of parameters between Mutian and MPV treatment groups for each disease type of FIP.

FIP	Parameters	Mutian Administration			MPV Administration			*p*-Value
		*n*	Median	Range	*n*	Median	Range	
Wet-type	Age (months)	43	7	4–156	14	6	2–186	0.3403
	Appetite score	43	4	1–6	14	4	1–5	0.7709
	Activity score	43	4	2–6	14	4	2–4	0.2506
	TB (mg/dL)	40	0.3	0.1–4.8	14	0.4	0.1–1.7	0.6245
Mixed-type	Age (months)	37	10	3–159	24	10	2–161	0.6250
	Appetite score	37	3	2–6	24	3	1–6	0.2820
	Activity score	37	3	2–5	24	3	2–6	0.1196
	TB (mg/dL)	37	0.4	0.1–5.4	24	0.3	0.1–4.7	0.2559
Dry-type	Age (months)	42	9	3–68	18	11	4–46	0.6857
	Appetite score	42	4	1–6	18	4	2–6	0.5989
	Activity score	42	4	1–6	18	4	1–6	0.2584
	TB (mg/dL)	33	0.1	0.1–2.7	18	0.2	0.1–3.6	0.7790

Abbreviations: MPV: molnupiravir; TB: total bilirubin; FIP: feline infectious peritonitis.

**Table 2:** Parametric statistical comparison of parameters between Mutian and MPV treatment groups for each disease type of FIP.

FIP	Parameters	Mutian Administration		MPV Administration		*p*-Value
		*n*	Mean ± SE	*n*	Mean ± SE	
Wet-type	Body temperature (°C)	43	39.13 ± 0.12	14	38.89 ± 0.21	0.2994
	SAA (μg/mL)	40	107.63 ± 10.40	14	116.84 ± 19.85	0.6752
Mixed-type	Body temperature (°C)	37	39.44 ± 0.13	24	39.22 ± 0.17	0.3154
	SAA (μg/mL)	37	122.74 ± 11.97	24	87.82 ± 11.27	0.0533
Dry-type	Body temperature (°C)	42	39.02 ± 0.11	18	38.96 ± 0.16	0.7540
	SAA (μg/mL)	42	95.68 ± 10.74	18	77.87 ± 18.07	0.3901

Abbreviations: MPV: molnupiravir; FIP: feline infectious peritonitis; SAA: serum amyloid-A; SE: standard error of the mean.

## Data Availability

The datasets used and/or analyzed in the current study are available from the corresponding author upon reasonable request.
